# ANCA-associated glomerulonephritis and lupus nephritis following COVID-19 vaccination: a case report and literature review

**DOI:** 10.3389/fimmu.2023.1298622

**Published:** 2024-01-08

**Authors:** Marcos Adriano Garcia Campos, Tiago de Oliveira Valois, Luís Eduardo Magalhães, Lucas Fernandes Vasques, Rafael Goulart de Medeiros, Denise Maria do Nascimento Costa, Natalino Salgado Filho, Raquel Moraes da Rocha Nogueira, Precil Diego Miranda de Menezes Neves, Gyl Eanes Barros Silva

**Affiliations:** ^1^ Clinical Hospital of State University Júlio de Mesquita Filho, State University of São Paulo, Botucatu, São Paulo, Brazil; ^2^ Divison of Nephrology, University Hospital of the Federal University of Maranhão, São Luís, Maranhão, Brazil; ^3^ Faculty of Medicine, University of Taubate, Taubaté, São Paulo, Brazil; ^4^ Divison of Nephrology, Hospital das Clínicas, Federal University of Pernambuco, Recife, Pernambuco, Brazil; ^5^ Recife Medical School, Federal University of Pernambuco, Recife, Pernambuco, Brazil; ^6^ Faculty of Medicine, CEUMA University, São Luís, Maranhão, Brazil; ^7^ Divison of Nephrology and Molecular Medicine, University of São Paulo, São Paulo, São Paulo, Brazil; ^8^ Department of Pathology and Legal Medicine, Ribeirão Preto Medical School, University of São Paulo, Ribeirão Preto, São Paulo, Brazil

**Keywords:** ANCA, glomerulonephritis, lupus nephritis, COVID-19, vaccine

## Abstract

With the coverage of COVID-19 vaccination, it has been possible to observe the potential side effects of SARS-CoV-2 vaccines, with the most common ones being fever, myalgia, headache, and fatigue. However, an association has been observed between new and recurrent kidney injuries, mainly glomerulonephritis and lupus nephritis associated with ANCA, with the Pfizer-BioNTech, Moderna, Sinovac, and AstraZeneca vaccines, although the relationship between them is not clear. We report a case of ANCA-related vasculitis and lupus glomerulonephritis after the second dose of the AstraZeneca vaccine. The elderly patient presented significant worsening of kidney function after immunosuppression and complications after a new onset COVID-19 infection that led to death. We provide a literature review about kidney damage related to ANCA vasculitis after COVID-19 vaccine, aiming for a better understanding of the pathophysiological mechanism of kidney injury, its presentation, and treatment.

## 1 Introduction

The most common side effects of the 2019 coronavirus vaccine (COVID-19) are fever, myalgia, headache, fatigue, local pain, and, rarely, anaphylactic reaction. However, case reports have demonstrated the occurrence of new and relapses cases of renal injury associated with SARS-CoV-2 immunization, including IgA nephropathy, membranous nephropathy, minimal change disease, anti-glomerular basement membrane disease, antineutrophil cytoplasmic antibody-associated vasculitis (ANCA), IgG4-related disease, lupus nephritis, scleroderma renal crisis, and thrombotic microangiopathy (1, 2). In these cases, such diseases had a temporal link with vaccination and a positive COVID-IgG response to SARS-CoV-2 (3).

Herein, we report a case from the Brazilian Consortium for the Study of COVID-19-Associated Renal Disease, which collects kidney biopsies from patients with kidney damage secondary to COVID-19 and its vaccine in 42 private and public hospitals (4, 5). This is a case report of ANCA-associated glomerulonephritis and lupus nephritis following COVID-19 vaccination, and a review of kidney manifestations related to this vaccine.

## 2 Case report

A 75-year-old woman presented to the emergency department with fever for 15 days, headache, adynamia, progressive dyspnea, polyarthralgia, macroscopic hematuria, foamy urine with no changes in urine volume. She had a previous diagnosis of hypothyroidism, hypertension and normal kidney function. She had been immunized against COVID-19 with the second dose AstraZeneca vaccine 15 days prior to admission. On physical examination, she was febrile (37.8 °C), normal blood pressure (130/80 mmHg), pulse rate 110 beats per minute, eupneic, and had an oxygen saturation of 98% in ambient air. The complementary physical examination was normal.

Three sequential RT-PCR tests for COVID-19 were negative, repeated every 2 days, and computed tomography (CT) of the chest showed no alterations. Laboratory tests on admission showed normocytic normochromic anemia, with hemoglobin (Hb) 10.4 g/dL and hematocrit (Ht): 30.1% and leukocytes: 10,810/mm3 with neutrophilia (9,015/mm3). Other laboratory tests were creatinine 2.9 mg/dL, sodium (Na): 128 mEq/L; potassium (K): 3.5 mEq/L, pH: 7.26, arterial partial oxygen pressure (PaO2): 81 mmHg; partial pressure of arterial carbon dioxide (PaCO2): 49 mmHg; bicarbonate: 20 mmol/L. Her urinalysis showed proteinuria, leukocyturia and hematuria, raising the hypothesis of nephritic syndrome. Her urine/protein/creatinine ratio was 2.5 mg/mg.

Although the RT-PCR test for COVID-19 was negative, the patient was admitted to the bed unit for COVID-19 cases for observation. Seven days after admission, her general condition worsened and her kidney function was compromised, with signs of hypervolemia such as peripheral edema, pulmonary congestion and dyspnea, requiring renal replacement therapy. At that time, laboratory tests showed Hb 7.5 g/dL; Ht 23.1%; platelets 104,000/mm3; D-dimer 3,250 ng/ml; ferritin 6,406 ng/ml; direct bilirubin 2.9 mg/dl; C-reactive protein 18.6 mg/dl; AST 230.6 U/L; ALT 139.4 U/L; urea: 105 mg/dl; Cr 2.7 mg/dl; procalcitonin: 0.98 ng/dl. Her c-ANCA serology was negative, with positive antinuclear antibody (ANA) (1:640) in a fine speckled pattern, as was p-ANCA (1:80). Considering the hypothesis of rapidly progressive glomerulonephritis, a kidney biopsy was performed five days after worsening (about 20 days after hospitalization). Laboratory findings at admission and seven days later are organized in **Table 1**.

**Table 1:** Laboratory findings on admission and seven days after admission.

Test	Value at admission	Value after 7 days	Normal ranges (female)
Hemoglobin (g/dL)	10.4	7.5	12.1 - 15.1
Hematocrit (%)	30.1	23.1	35 - 45
Platelets (cells/mm³)		104,000	250,000 - 260,000
Leukocytes (cells/mm³)	10,810		4,500 - 11,000
Neutrophils (cells/mm³)	9,015		1,500 - 7,000
Urea (mg/dL)		105	21 - 43
Creatinine (mg/dL)	2.9	2.7	0.6 - 1.1
Sodium (mEq/L)	128		135 - 145
Potassium (mEq/L)	3.5		3.5 - 5.5
AST (IU/L)	27	231	10 - 36
ALT (IU/L)	24	139	4 - 36
GGT (IU/L)		44	05 - 43
D-dimer (ng/mL)		3,250	< 250
Ferritin (ng/mL)		6,406	11 - 306
Direct bilirubin (mg/dL)		2.9	< 1
Indirect bilirubin (mg/dL)		1.0	< 0.5
C-reactive protein (mg/dL)		18.6	< 0.3
Procalcitonin (ng/dL)		0.98	< 200
Blood pH level	7.26		7.35 - 7.45
Arterial partial oxygen pressure (mmHg)	81		80 - 100
Partial pressure of arterial carbon dioxide (mmHg)	49		35 - 45
Bicarbonate (mmol/L)	20		23–28
Urine/protein creatinine ratio (mg/g)	2.5		< 150

^AST, Aspartate transaminase; ALT, Alanine transaminase; GGT, Gamma-glutamyl transferase^

**Figure 1:**
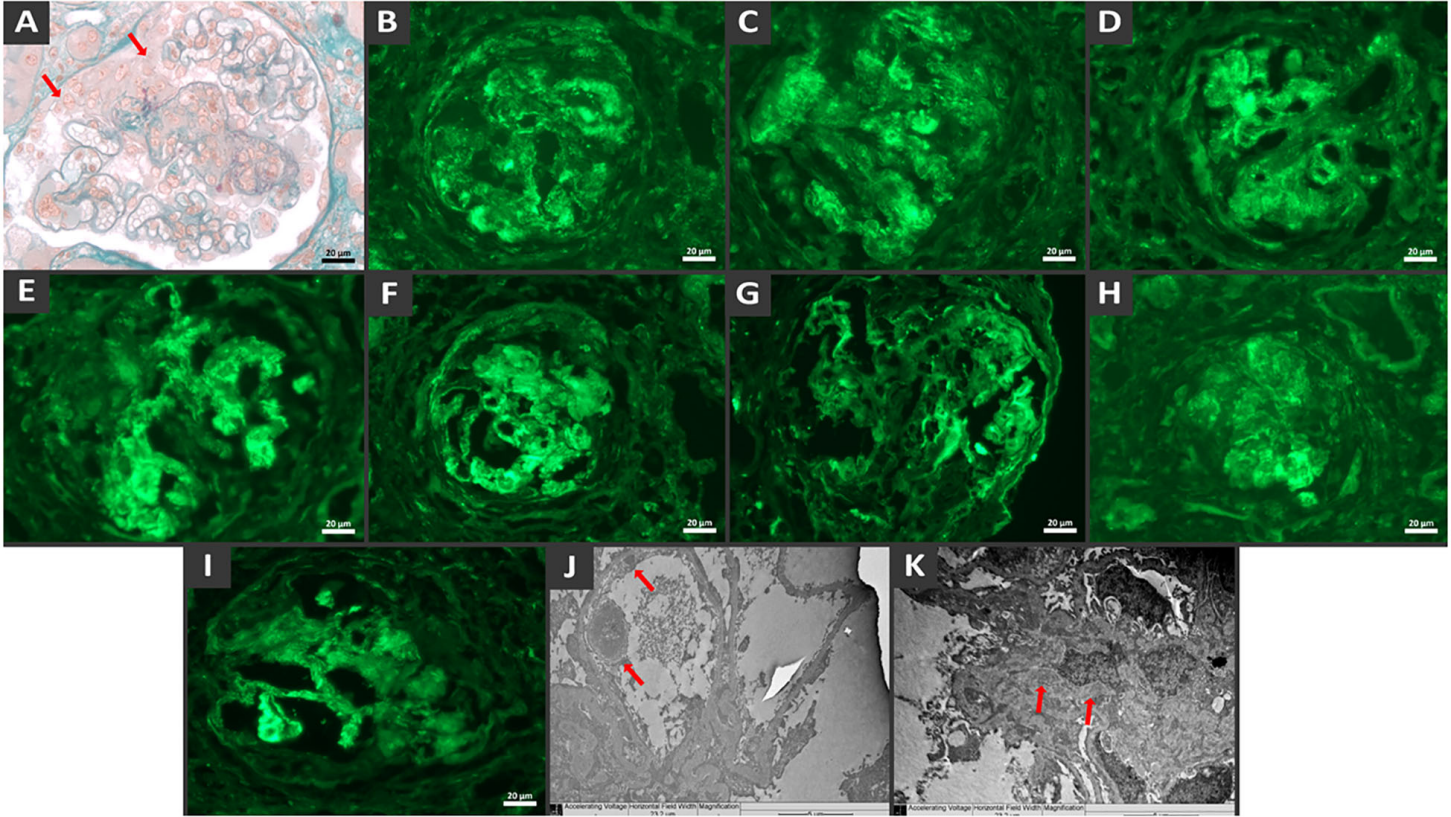
Histological findings show diffuse crescentic necrotizing glomerulonephritis with endocapillary proliferation and full-house immune complex deposition. Glomerulus with cellular crescent (arrow), endocapillary crescent and fibrinoid necrosis by light microscopy, Masson’s trichrome stain **(A)**. Direct immunofluorescence demonstrates granular mesangial and capillary wall staining deposits of IgG **(B)**, IgM **(C)**, IgA **(D)**, C3 **(E)**, C1q **(F)**, Fibrinogen **(G)**, Kappa **(H)** e Lambda **(I)**. Note the crescent surrounding the vascular tuft. Electron microscopy from paraffin embedded tissue reveals electron-dense deposits in the subendothelial **(J)** and mesangial **(K)** regions.

A renal biopsy revealed a proliferative and necrotizing glomerulonephritis with a full house immunofluorescence pattern, leading to the suspicion of lupus nephritis (**Figure 1**). There was mild interstitial fibrosis, tubular atrophy and degeneration of the tubular epithelium. The blood vessels showed no significant changes. Because of the presence of diffuse crescents and the necrotizing appearance of the lesion, the histological hypothesis of lupus glomerulonephritis associated with ANCA-related vasculitis was confirmed.

**Figure 2:**
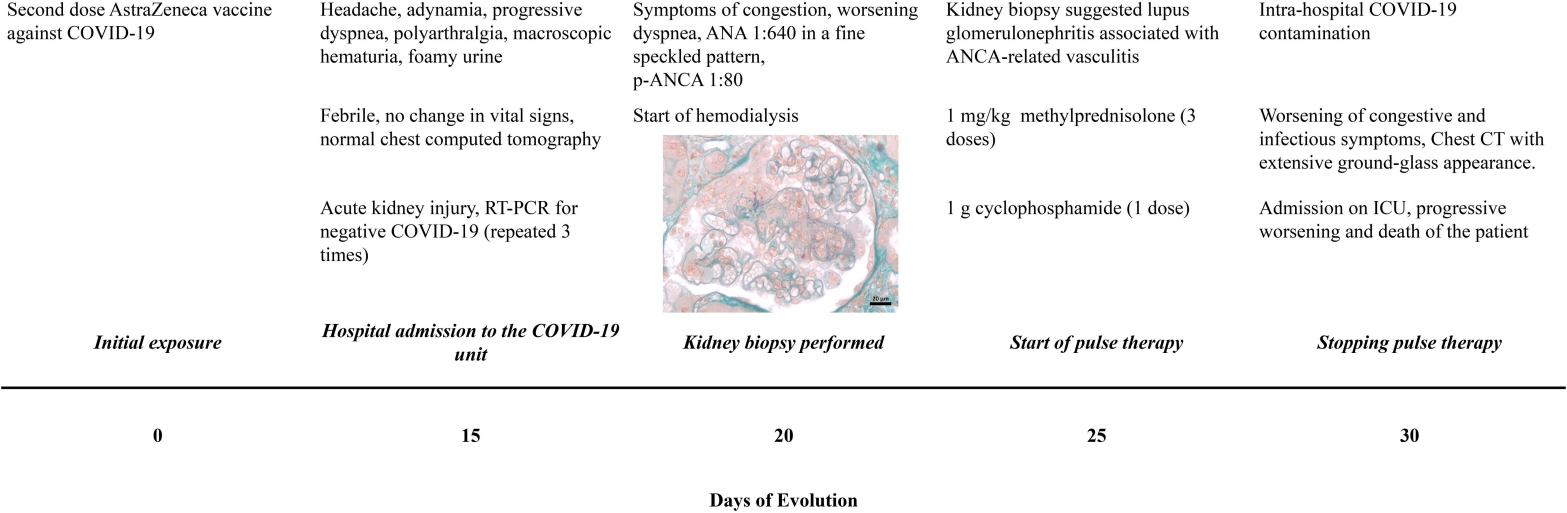
Timeline of clinical evolution. The time and initial symptoms are specified from COVID-19 vaccination, investigation, treatment and patient outcome.

The patient was treated methylprednisolone 500mg for 3 days, followed by prednisone 1 mg/kg/day, and administered one dose of cyclophosphamide 1 g. After 30 days of the first symptoms, the RT-PCR test for COVID-19 was positive, possibly due to intra-hospital contamination. The imunossupression drugs were stopped. Immunoglobulin, plasmapheresis or remdesivir was not performed. Her general condition worsened rapidly, requiring intensive care due to dyspnea. Chest CT showed a extensive bilateral pleural effusion extensive with laminar consolidations, adjacent atelectasis, diffuse bilateral ground-glass opacities involving 75% of the parenchym, and signs of parapseptal and centrilobular emphysema. Unfortunately, the patient presented clinical worsening, with refractory hemodynamic instability, respiratory acidosis, and hypoxia, and evolved to death. The timeline of the patient’s evolution is shown in **Figure 2**.

## 3 Discussion

The remarkable increase in the coverage of immunization against COVID-19 worldwide allows us to learn about the possible adverse effects of the vaccine, including those involving the renal system. Some vaccines have been previously linked to kidney injury. The influenza vaccine has been associated with nephrotic syndrome secondary to minimal change disease (6, 7), membranous nephropathy (8), vasculitis with pauci-immune glomerulonephritis (9), microscopic polyangiitis (10), ANCA-associated glomerulonephritis (9), rhabdomyolysis with acute kidney injury (11), vasculitis (12) and Henoch-Schonlein purpura (13, 14). The hepatitis B vaccine has been associated with nephrotic syndrome secondary to minimal change disease (15) and lupus nephritis (16). The pneumococcal vaccine was associated with glomerulonephritis (related to anti-glomerular basement membrane antibody) (17), the polio-diphtheria-tetanus vaccine with nephrotic syndrome (secondary to minimal change disease) (18), the varicella vaccine with nephrotic syndrome (19), and the measles vaccine with minimal change disease (20). The rabies vaccine was associated with possible relapse of nephrotic syndrome (21). The Bacillus Calmette-Guerin (BCG) vaccine used as intravesical immunotherapy to treat bladder cancer and not for immunization was associated with membranous nephropathy, interstitial nephritis, and formation of asymptomatic renal granulomas (22).

Several COVID-19 vaccines and their different mechanisms of action have been reported to have some renal injury side effects (4) (**Table 2**). Lipid-based nanoparticle-mRNA vaccines cause a stimulatory response to CD4+ and CD8+ T lymphocytes and an increased production of B lymphocytes in germinal centers, resulting in the secretion of interferons (mainly gamma) and interleukin-2. DNA-adenovirus vaccines stimulate CD4+ and CD8+ cytotoxic T lymphocytes, in addition to increasing antibody production by B lymphocytes, mainly IgG1-IgG3 and to a lesser extent IGg2-IgG4 (2). Both vaccine classes have the same antigenic target, the viral spike protein, and have been associated with glomerular injury.

**Table 2:** New onset and relapses cases of ANCA-associated glomerulonephritis and other glomerular lesions with ANCA-positive association reported after COVID-19 vaccination.

Cases of renal involvement reported after COVID-19 vaccination	Total number of cases reported	Type of vaccine	Number of Cases per dose
ANCA-associated Glomerulonephritis^a^	31*	mRNA Vaccine - 17 Viral Vector Vaccine - 10 Inactivated Vaccine - 4	1st dose: 9 2nd dose: 19 3nd dose: 3
Lupus Nephritis (2* cases ANCA-positive)^b^	6*	mRNA Vaccine - 3 Viral Vector Vaccine - 3	1st dose: 4 2nd dose: 2
IgA Nephropathy (3 cases ANCA-positive)^c^	30	mRNA Vaccine - 28 Viral Vector Vaccine - 1 Inactivated Vaccine - 1	1st dose: 5 2nd dose: 25
Anti-glomerular basement membrane nephritis (6 cases ANCA-positive)^d^	14	mRNA Vaccine - 13 Viral Vector Vaccine - 1	1st dose: 5 2nd dose: 8 3nd dose: 1
Crescentic glomerulonephritis (6 cases ANCA-positive)^e^	7	mRNA Vaccine - 7	1st dose: 1 2nd dose: 6
Membranous nephropathy (1 case ANCA-positive)^f^	7	mRNA Vaccine - 6 Viral Vector Vaccine - 1	1nd dose: 2 2nd dose: 5
Other non-ANCa-positive glomerular lesions			
Minimal change disease^g^	22	mRNA Vaccine - 20 Viral Vector Vaccine - 2	1st dose: 6 2nd dose: 8
Collapsing glomerulopathy^h^	4	mRNA Vaccine - 2 Viral Vector Vaccine - 2	1st dose: 2 2nd dose: 2

ANCA: anti-neutrophil cytoplasmic antibodies.

*Including the present case.

References: ^a^ (23–47); ^b^ (48–52); ^c^ (47, 48, 53–61); ^d^ (47, 56, 62–68); ^e^ (48, 69); ^f^ (47–49, 70); ^g^ (47, 48, 53, 71–85); ^h^ (2, 86, 87).

The pathophysiological mechanism of post-vaccine glomerulonephritis remains unknown. However, the likely cause is mimicry of the remade viral proteins with the host’s proteins, thereby causing a type of “second wave infection” and subsequent renal damage (1). Infection by SARS-CoV-2 has been recognized as a trigger for the onset of several diseases and autoimmune reactions (48). The viral RNA is recognized by host receptors, binding to them and stimulating the production of type I interferon and inflammatory cytokines. In turn, type I interferon stimulates antibodies production, which are strictly associated with autoimmune diseases (1, 2).

The SARS-CoV-2 vaccine seems to be related to some cases of ANCA-associated glomerulonephritis and lupus nephritis, majority related after Pfizer-BioNTech, Moderna, Sinovac and AstraZeneca (23). The relationship between the vaccine and autoimmune diseases is still unclear. The mRNA vaccines can induce, for example, double-positive anti–glomerular basement membrane antibody and myeloperoxidase ANCA (62), subsequently nephritis and vasculitis. Proinflammatory status mediated by the vaccine containing lipid-based nanoparticles can lead to a subsequent loss of tolerance to self-antigens due to autoreactivity and immune system hyperactivation (88). It is suggested that the vaccines’ antigenic target, the viral spicule protein, stimulates antibody production.

The overlap of ANCA-associated glomerulonephritis and lupus nephritis is not uncommon (89). Although these diseases are easy to differentiate from each other by autoantibody profile and histopathological findings, some patients have shown an overlap of such findings (90). Crescentic glomerulonephritis is not rare in lupus nephritis, and the presence of ANCA antibodies is also well established in the literature (90, 91). ANCA antibodies are present more markedly in crescentic lupus nephritis than in lupus nephritis without crescents; such a finding favors the role of ANCA antibodies in renal crescent formation. Similarly, although associated p-ANCA glomerulonephritis is defined as pauci-immune on immunofluorescence, the presence of immune-complexes described in the present case is explained by the full house pattern of overlapping lupus nephritis (90, 91).

We present **Supplementary Material** with a detailed review of the literature on ANCA-related glomerulonephritis and other renal lesions in which ANCA positivity occurred after COVID-19 vaccination. The main conditions were ANCA glomerulonephritis (14%), IgA nephropathy (12.3%), anti-glomerular basement membrane glomerulonephritis (10.5%), crescentic glomerulonephritis (10.5%). In this review, 55.17% were women, 81.03% were vaccinated with the mRNA vaccine. The symptoms were nonspecific and started on average 18 days after the vaccine. Most patients used high doses of glucocorticoids and some type of immunosuppressant, and 18.96% required hemodialysis. In general, clinically significant chronic kidney disease may persist in more than 75% of ANCA-associated vasculitis in those with kidney impairment (24). The proportion of normal glomeruli appears to be associated with dialysis discontinuation, although some treatments such as plasmapheresis did not show significance with dialysis discontinuation (25).

## 4 Conclusion

The study of adverse effects, including renal involvement, of the COVID-19 vaccine, gives us the opportunity for early diagnosis and provision of assistance to patients with complications after autoimmune activation by the vaccine or direct contact with the SARS-CoV-2 virus.

## Data availability statement

The original contributions presented in the study are included in the article/**Supplementary Material**. Further inquiries can be directed to the corresponding author.

## Ethics statement

The requirement of ethical approval was waived by Comitê de Ética e Pesquisa do HUUFMA for the studies involving humans because Comitê de Ética e Pesquisa do HUUFMA. The studies were conducted in accordance with the local legislation and institutional requirements. The participants provided their written informed consent to participate in this study. Written informed consent was obtained from the individual(s) for the publication of any potentially identifiable images or data included in this article.

## Author contributions

MC: Conceptualization, Writing – original draft. TV: Data curation, Investigation, Writing – original draft. LM: Data curation, Investigation, Writing – original draft. LF: Data curation, Investigation, Writing – original draft. RM: Data curation, Investigation, Writing – original draft. DC: Writing – review & editing. NS: Resources, Writing – review & editing. RN: Conceptualization, Investigation, Writing – original draft. PN: Writing – review & editing. GS: Supervision, Writing – review & editing.
