# Supplementary material for: ANCA-associated glomerulonephritis and lupus nephritis following COVID-19 vaccination: a case report and literature review
Source: Front Immunol. 2024 Jan 8;14:1298622. doi: 10.3389/fimmu.2023.1298622 (PMC10828972; doi:10.3389/fimmu.2023.1298622)
Supplement: Supplementary file 1 [file DataSheet_1.pdf]

ANCA-ASSOCIATED GLOMERULONEPHRITIS AND LUPUS NEPHRITIS FOLLOWING COVID-19 VACCINATION: A CASE REPORT AND LITERATURE REVIEW

Supplementary Table. Review of literature cases of kidney injury after COVID-19 vaccine with ANCA positivity.

Case (Reference)	Age	Sex	Race	Comorbidities	Diagnosis	New Onset or Recurrence	Vaccine	Dose	Onset Time (days)	Presenting Symptoms	Kidney Biopsy Findings	Baseline Creatinine (mg/dl)	Initial Creatinine (mg/dl)	ANCA Type	Follow-up (weeks)	Treatment	Response	Renal Replacement Therapy
#1 (30)	81	Male	-	None	IgAN	NO	Moderna	2	-	Flu-like symptoms	-	-	-	PR3-ANCA	3	High-dose glucocorticoids, cyclophosphamide	Recovery	Plasmapheresis
#2 (31)	52	Male	White	SAH	Pauci-immune necrotizing and crescentic glomerulonephritis	NO	Moderna	2	1	Headache and weakness	Cellular crescents, fibrinoid necrosis, tubular injury, IFTA	1.11	8.41	PR3-ANCA	2	Rituximab, cyclophosphamide, prednisone	Chronic hemodialysis	Hemodialysis
#3 (32)	29	Female	-	Congenital diffuse cystic lung disease	Pauci-immune crescentic glomerulonephritis	NO	Pfizer	2	49	-	Glomerular sclerosis, cellular crescents, fibrocellular crescents, diffuse mononuclear interstitial inflammation, foci of moderate tubulitis, and scattered red blood cell casts, focal FPE	0.8	1.91	MPO-ANCA	12	Methylprednisolone, rituximab, cyclophosphamide	Recovery	
#4 (33)	70	Female	-	None	Pauci-immune crescent glomerulonephritis	NO	Moderna	1	7	Dizziness, headache, and hematuria	Acute severe renal vasculitis	1	3.5	MPO-ANCA	3	High-dose glucocorticoids, anti-CD20 therapy	Recovery	Plasmapheresis
#5 (34)	54	Female	Caucasian	Seronegative polyarthritis	Chronic pauci-immune necrotising glomerulonephritis	NO	Pfizer	2	42	Weak and complained of dizziness and loss of appetite	Tuft necroses and fibrocellular crescents	1.6	2.11	MPO-ANCA	6	Methylprednisolone, rituximab	Recovery	
#6 (34)	78	Female	Caucasian	Microscopic polyangiitis	Chronic pauci-immune necrotising glomerulonephritis	Rec	Pfizer	2	56	Dark urine, arthralgia, fatigue, and nausea	-	-	8.23	MPO-ANCA	-	Methylprednisolone, rituximab	Chronic hemodialysis	Hemodialysis
#7 (35)	58	Male	Caucasian	None	Anti-PR3-associated ANCA glomerulonephritis	NO	Moderna	2	56	Nausea, vomiting, and a loss weight	Pauci-immune necrotizing and CrGN	-	4.1	C-ANCA, PR3-ANCA	10	Prednisone, cyclophosphamide, rituximab	Recovery	Plasmapheresis
#8 (36)	79	Female	Caucasian	SAH, degenerative disc disease	MPO-positive AAV	NO	Pfizer	2	14	Weakness and upper thigh pain	Severe acute tubular injury with pauci-immune crescentic GN and interstitial nephritis	0.71	6.57	MPO-ANCA	-	Methylprednisolone, prednisone, cyclophosphamide	Recovery	
#9 (38)	78	Female	-	DM, SAH, atrial fibrillation	MPO-positive AAV	NO	Pfizer	2	28	Nausea, vomiting, diarrhea and lethargy	Crescent necrotizing GN with moderate interstitial inflammation	0.77	3.54	MPO-ANCA	4	Methylprednisolone, Prednisone	Recovery	
#10 (39)	63	Male	-	None	ANCA-associated pauci-immune glomerulonephritis	NO	Oxford-AstraZeneca	1	7	Flu-like symptoms, haemoptysis	Focal extracapillary proliferation and crescent formation	-	2.90	MPO-ANCA	6	High-dose glucocorticoids, prednisone, cyclophosphamide	Chronic hemodialysis	Hemodialysis
#11 (40)	75	Male	-	Microscopic polyangiitis	AAV relapse	Rec	Oxford-AstraZeneca	1	35	Haemoptysis	Pauci-immune crescentic GN	-	-	MPO-ANCA	-	Methylprednisolone, rituximab	Chronic hemodialysis	Hemodialysis
#12 (40)	74	Male	-	Microscopic polyangiitis	AAV relapse	Rec	Oxford-AstraZeneca	2	14	-	GN, consistent with AAV	-	9.97	MPO-ANCA	-	Methylprednisolone, cyclophosphamide	Recovery	Hemodialysis
#13 (41)	78	Female	White	None	AAV	NO	Sinovac	2	14	Fever, fatigue, weight loss, night sweats, and renal dysfunction	Necrotizing and CrGN	0.8	7.3	PR3-ANCA	12	Corticosteroids, cyclophosphamide	Recovery	Hemodialysis
#14 (42)	47	Female	-	None	ANCA-GN	NO	Pfizer	1	-	Bilateral flank pain, generalized weakness, and bilateral lower extremity swelling	Fibrous crescents with interstitial fibrosis, IFTA	0.8	2.91	MPO-ANCA	12	Methylprednisolone , prednisone, azathioprine	Recovery	
#15 (43)	72	Female	-	None	ANCA-GN	NO	Moderna	3	18	Anorexia, abdominal pain, and febrile sensation	Fibrocellular crescents, GG, moderate IFTA	0.8	4.71	MPO-ANCA	2	Methylprednisolone, cyclophosphamide	Recovery	Plasmapheresis
#16 (44)	62	Female	-	Systemic sclerosis, interstitial lung disease, DM	ANCA-GN	NO	Pfizer	2	60	Asymptomatic	CrGN	<1	5.18	MPO-ANCA	32	Methylprednisolone, cyclophosphamide, prednisone	Recovery	
#17 (45)	84	Male	-	Cerebral infarction, colon cancer	Renal-limited MPO-AAV	NO	Pfizer	2	14	Fever, malaise, cervical lymphadenopathy and cough	Focal necrotizing glomerulonephritis with cellular crescents	1.17	1.22	MPO-ANCA	8	Methylprednisolone, prednisolone	Recovery	
#18 (46)	51	Male	-	None	PR3-AAV	NO	Oxford-AstraZeneca	1	15	Low-grade fever with debilitating inflammatory	Pauci-immune CrGN	1.2	4.8	PR3-ANCA	20	Prednisolone, rituximabe	Recovery	
#19 (47)	15	Male	-	None	ANCA-GN	NO	Sinopharm	2	-	Colicf-like non-positional pain of both flanks, flu-like symptoms, nausea, darkening of urine color and loss of appetite	Pauci-immune crescentic and necrotizing GN and mild tubulointerstitial nephritis with foci of acute tubular injury	-	3	MPO-ANCA	-	Prednisolone, mycophenolate mofetil	Recovery	
#20 (48)	42	Male	-	None	ANCA-GN	NO	Pfizer	2	14	General weakness, edema, shortness of breath, edema, gross hematuria, and significant weight loss	Diffuse necrotising and CrGN with mild interstitial inflammation	0.97	3.05	MPO-ANCA	33	Methylprednisolone, prednisolone, rituximab	Recovery	Plasmapheresis
#21 (49)	72	Male	-	Prostatic hipertrofy	ANCA-GN	NO	Pfizer	2	21	Fever, fatige, loss of apetite	Cellular crescents, segmental necrosis, and destruction of the Bowman's capsule	-	5.0	MPO-ANCA	1	Methylprednisolone, prednisolone, rituximab	Recovery	Hemodialysis
#22 (50)	52	Male	-	SAH, uterine malignancy	ANCA-GN	NO	Johnson	1	12	Fever, joint pain, weakness of all limbs	Necrotizing and CrGN	0.8	6.3	c-ANCA, p-ANCA	1	Cyclophosphamide	Recovery	

#23 (51)	58	Female	-	Hypertireoidism	RPGN	NO	Oxford-AstraZeneca	1	5	Fatigue, paleness, arthralgia on hands, knees, ankles, foamy urine, and elevated blood pressure	CrGN with glomerular sclerosis, fibrous crescents, interstitial fibrosis, IFTA, inflammatory lesions, endothelial swelling, endocapillary proliferation, accumulation of macrophages, hyaline deposits and cellular and fibrocellular crescents	1.0	2.2	p-ANCA	42	Methylprednisolone, cyclophosphamide, azathioprine	Recovery	
#24 (52)	67	Female	Asian	SAH	RPGN	NO	Covaxin	2	7	Nausea, decrease in appetite, increase in swelling, and breathlessness	Crescents and necrotizing lesion with fibrinoid necrosis in crescentic glomeruli, moderate IFTA	1.2	6.4	MPO-ANCA	4	Methylprednisolone, cyclophosphamide	Recovery	Hemodialysis
#25 (24)	76	Female	-	DM, SAH, dyslipidemia	ANCA-GN	NO	Oxford-AstraZeneca	2	11	Chronic fever, weight loss, and productive cough	Fibrinoid necrosis of arteries and severe tubulointerstitial inflammation, IFTA	1.5	1.7	p-ANCA	1	Cyclophosphamide	Recovery	Hemodialysis
#26 (24)	69	Female	-	SAH	AAV with ANCA-GN	NO	Oxford-AstraZeneca + Pfizer	2	30	Anorexia, fatigue, a weight loss and mixed conductive and sensorineural hearing loss	Fibrinoid necrosis was found on the vasculitic medium-sized artery, IFTA	0.7	2.4	p-ANCA	32	Methylprednisolone, cyclophosphamide	Recovery	
#27 (24)	84	Female	-	SAH, dyslipidemia	ANCA-GN with eosinophilic interstitial nephritis	NO	Oxford-AstraZeneca + Pfizer	1	7	Fever, drowsiness, progressive dysphagia, decreased urine volume, and a weight loss	Necrosis fibrinoide, diffuse tubulointerstitial inflammation with abundant eosinophils and trivial IFTA	0.9	5.1	c-ANCA	2	Methylprednisolone, immunoglobulin	Recovery	Hemodialysis
#28 (53)	38	Male	White	-	IgAN	NO	Pfizer	2	14	Gross hematuria	-	1.3	1.6	p-ANCA	-	Conservative	-	
#29 (53)	44	Male	White	-	IgAN and ATN	NO	Moderna	2	14	AKI	-	1.1	2.5	p-ANCA	12	High-dose glucocorticoids, prednisone, cyclophosphamide	Not Recovery	
#30 (53)	66	Male	White	-	IgAN	NO	Moderna	2	14	Gross hematuria	-	1.1	1.5	p-ANCA	20	Prednisone	Recovery	
#31 (53)	62	Male	White	-	IgAN	NO	Pfizer	6	42	AKI	-	1	2.2	p-ANCA	6	Conservative	Recovery	
#32 (53)	77	Male	White	-	Atypical anti-GBM nephritis	NO	Pfizer	1	7	Hypertension	-	1	1.8	p-ANCA	6	Prednisone, mycophenolate mofetil		
#33 (53)	83	Male	White	-	MCD and ATN	NO	Moderna	4	28	AKI	-	1.19	2.19	p-ANCA	4	High-dose glucocorticoids	Recovery	
#34 (53)	50	Female	White	-	NELL-1 MN	NO	Pfizer	4	28	Joint pain and proteinuria	-	0.84	0.7	p-ANCA	8	Conservative	Recovery	
#35 (53)	82	Female	White	-	MPO-ANCA	NO	Moderna	4	28	AKI, hematuria, proteinuria	-	0.8	2.5	p-ANCA	4	High-dose glucocorticoids, rituximab	Recovery	
#36 (53)	67	Female	White	-	MCD	NO	Moderna	3	21	Edema	-	1	1.6	p-ANCA	8	High-dose glucocorticoids, rituximab	Recovery	
#37 (53)	29	Female	Asian	-	FSGS (tip-variant)	NO	Pfizer	3	21	Edema	-	0.6	0.6	p-ANCA	10	High-dose glucocorticoids, tacrolimus	Recovery	
#38 (53)	39	Male	White	-	PLA2R MN	NO	Pfizer	1	7	Edema	-	0.91	1.13	p-ANCA	4	Tacrolimus	Recovery	
#39 (53)	70	Male	White	-	PLA2R MN	NO	Moderna	4	28	Edema	-	1.7	2.1	p-ANCA	-	Obinutuzumab	-	
#40 (53)	19	Male	White	-	IgAN	NO	Moderna	1	7	Gross hematuria	-	0.96	0.76	p-ANCA	-	Conservative	-	
#41 (23)	16	Female	Hispanic	-	Diffuse LN	NO	Pfizer	1	2	Gross hematuria	MME, mesangial and endocapillary hypercellularity	-	0.7	p-ANCA	2	Methylprednisolone, prednisone	Recovery	
#42 (23)	72	Female	Hispanic	-	IgAN	NO	Pfizer	2	2	Hematuria, AKI	MME, mesangial hypercellularity, ATI, necrosis, crescents, SS, moderate IFTA	-	4.9	p-ANCA	2	Methylprednisolone, prednisone, cyclophosphamide	Not Recovery	
#43 (23)	40	Female	White	-	IgAN	NO	Moderna	2	7	Hematuria	MME, ATIN, moderate AH and MD	-	1.3	p-ANCA	2	Conservative	Not Recovery	
#44 (66)	41	Female	Asian	Gestational DM	IgAN	NO	Pfizer	2	1	Headache, generalized myalgia, hypertension and gross hematuria	CRGN, mild tubulointerstitial inflammation. mild IFTA, mild AH	-	1.73	p-ANCA	-	Methylprednisolone, prednisolone, cyclophosphamide	Recovery	
#45 (25)	63	Female	White	Asthma, macular degeneration, cataract	anti-GBM GN	NO	Pfizer	3	7	-	Severe acute tubulointerstitial nephritis, mildI IFTA, GG	-	7.09	MPO-ANCA	44	Conservatives, Cyclophosphamide, Plasmapheresis	Recovery	Plasmapheresis
#46 (25)	51	Male	Indigenous	Polysubstance use disorder, SAH; heart failure with reduced ejection fraction	anti-GBM GN	NO	Pfizer	2	14	-	Severe ATIN, minimal interstitial fibrosis and tubular atroph, GG	-	3.14	MPO-ANCA	44	High-dose glucocorticoids, cyclophosphamide, plasmapheresis	Not Recovery	Plasmapheresis
#47 (25)	62	Male	Hispanic	Diverticulosis, vitiligo, ex-smoker	anti-GBM GN	NO	Pfizer	1	21	-	Mild IFTA, GG	-	7.32	MPO-ANCA	16	High-dose glucocorticoids, cyclophosphamide, plasmapheresis	Not Recovery	Plasmapheresis
#48 (25)	70	Female	White	Hypothyroidism, recent myocardial infarction, SAH; dyslipidemia; DM ex-smoker	anti-GBM GN	NO	Moderna	3	42	-	Moderate ATIN, minimal IFTA, GG	-	6.79	MPO-ANCA	-	High-dose glucocorticoids, cyclophosphamide, plasmapheresis	Recovery	Plasmapheresis
#49 (68)	74	Female	-	Chronic kidney disease, nontuberculous mycobacterial infection, ex-smoker	anti-GBM GN	NO	Pfizer	2	2	Persistent fever and malaise	CrGN with moderate interstitial inflammation	0.85	1.28	MPO-ANCA	73	Rituximab	Recovery	

#50 (70)	23	Male	Hispanic	Fragile X syndrome, interstitial lung disease of unclear etiology	anti-GBM GN	NO	Moderna	2	14	Weakness, fatigue, and weight loss	Diffuse CrGN	0.7	14	MPO-ANCA	-	-	-	
#51 (23)	76	Male	White	-	CrGN	NO	Pfizer	2	2	Hematuria, AKI	Necrosis, crescents, severe AS, mild IFTA, severe FPE	-	8.6	p-ANCA	3	Methylprednisolone, prednisone, rituximab	Not Recovery	Hemodialysis
#52 (23)	81	Female	White	-	CrGN	NO	Pfizer	2	5	Hematuria, AKI	MME, crescents, SS, ATIN, mild IFTA, severe AS, mild AH, mild FPE	-	3.1	p-ANCA	3	Rituximab	Not Recovery	
#53 (23)	76	Female	White	-	CrGN	NO	Moderna	1	14	Hematuria, AKI	Necrosis, crescents, ATIN, mild IFTA, severe AS, moderate AH, severe FPE	-	3	p-ANCA	5	Methylprednisolone, prednisone, rituximab	Parcial recovery	
#54 (23)	71	Female	White	-	CrGN	NO	Moderna	2	14	Hematuria	ATIN, moderate IFTA, severe AS, MD, moderate FPE	-	1.3	p-ANCA	1	Methylprednisolone, prednisone, rituximab	Not Recovery	
#55 (23)	65	Female	White	-	CrGN	NO	Pfizer	2	14	Hematuria	Necrosis, crescents, ATIN, moderate IFTA	-	3.29	p-ANCA	2	Methylprednisolone, prednisone, cyclophosphamide	Not Recovery	
#56 (23)	79	Female	White	-	CrGN	Rec	Moderna	2	21	Hematuria	Necrosis, crescents, SS, ATIN, mild IFTA	-	1.12	p-ANCA	16	Rituximab	Recovery	
#57 (23)	54	Male	Asian	-	MN	NO	Moderna	2	1	Hematuria	Crescents, SS, ATIN, moderate AH, MD, subepithelial deposits, severe FPE	-	1.3	p-ANCA	8	Methylprednisolone, prednisone, rituximab	Not Recovery	
#58 (article case)	75	Female	White	Hypothyroidism, SAH	ANCA-GN and LN	NO	Oxford-AstraZeneca	2	15	Fever, gross hematuria, headache, weakness	Necrotizing glomerulonephritis with a full house immunofluorescence pattern	<1	2.9	p-ANCA	40	Methylprednisolone, cyclophosphamide	Not Recovery	

AAV: ANCA associated vasculitis; AH: arteriolar hyalinosis; AKI: acute kidney injury; ANCA: antineutrophil cytoplasmic antibodies; AS: arterio sclerosis; ATIN: acute tubulointerstitial nephritis; ATN: acute tubular necrosis; CrGN: crescentic glomerulonephritis; DM: diabetes mellitus; FPE: foot process effacement; FSGS: focal segmental glomerulosclerosis; GBM: glomerular basement membrane; GG: global glomerulosclerosis; GN: glomerulonephritis; IFTA: interstitial fibrosis/tubular atrophy; IgAN: IgA nephropathy; LN: lupus nephritis; MCD: minimal change disease; MD: mesangial deposits; MME: mesangial matrix expansion; MN: membranous nephropathy; MPO: myeloperoxidase; NELL-1: Neural epidermal growth factor-like 1 protein; NO: new onset; PLA2R: anti-phospholipase A2 receptor; PR3: proteinase 3; Rec: recurrence; RPGN: Rapidly progressive glomerulonephritis; SAH: systemic arterial hypertension; SS: segmental sclerosis
