# Supplementary material for: ANCA-associated glomerulonephritis and lupus nephritis following COVID-19 vaccination: a case report and literature review
Source: Front Immunol. 2024 Jan 8;14:1298622. doi: 10.3389/fimmu.2023.1298622 (PMC10828972; doi:10.3389/fimmu.2023.1298622)
Supplement: Supplementary file 2 [file DataSheet_2.pdf]

Search terms

Bibliographic search was carried in PUBMED/MEDLINE, PUBCOVID19, and GOOGLE SCHOLAR databases. The keywords related to “Glomerular”, “Glomerulopathy”, “Kidney”, “Tubular”, “proteinuria,” and “COVID-19”, “SARS-CoV-2”, and “SARS-CoV vaccine” were used with Boolean combination
