# Supplementary figures and images for: ANCA-associated glomerulonephritis and lupus nephritis following COVID-19 vaccination: a case report and literature review
Source: Front Immunol. 2024 Jan 8;14:1298622. doi: 10.3389/fimmu.2023.1298622 (PMC10828972; doi:10.3389/fimmu.2023.1298622)

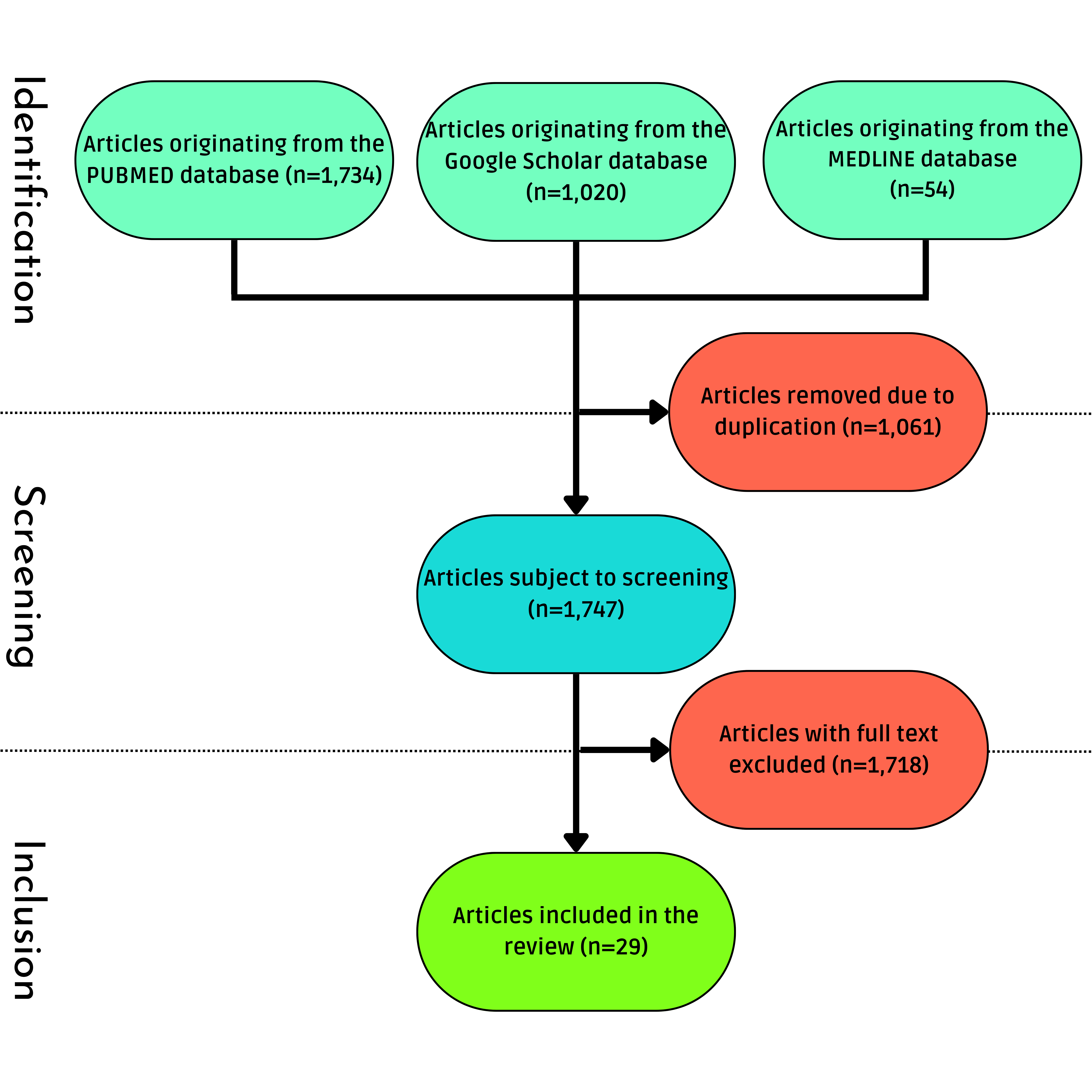

Supplement: Supplementary file 3 [file Image_1.png]
